# Relationship between tonic inhibitory currents and phasic inhibitory activity in the spinal cord lamina II region of adult mice

**DOI:** 10.1186/1744-8069-2-36

**Published:** 2006-11-27

**Authors:** Toyofumi Ataka, Jianguo G Gu

**Affiliations:** 1 Department of Oral and Maxillofacial Surgery, McKnight Brain Institute and College of Dentistry, University of Florida, Gainesville, Florida 32610, USA; 2 Division of Anesthesiology, Niigata University Graduate School of Medical and Dental Sciences, Niigata 951-8510, Japan

## Abstract

Phasic and tonic inhibitions are two types of inhibitory activities involved in inhibitory processing in the CNS. In the spinal cord dorsal horn, phasic inhibition is mediated by both GABAergic and glycinergic inhibitory postsynaptic currents. In contrast to phasic inhibitory currents, using patch-clamp recording technique on spinal cord slices prepared from adult mice we revealed that tonic inhibitory currents were mediated by GABA_A _receptors but not by glycine receptors in dorsal horn lamina II region. We found that there was a linear relationship (r = 0.85) between the amplitude of tonic inhibitory currents and the frequency of GABAergic inhibitory postsynaptic currents. Analysis of charge transfer showed that the charges carried by tonic inhibitory currents were about 6 times of charges carried by phasic inhibitory currents. The prominent charge transfer by tonic inhibitory currents and their synaptic activity dependency suggest a significant role of tonic inhibition in sensory processing.

## Background

GABA (γ-Aminobutyric acid) and glycine are two principle inhibitory neurotransmitters in the spinal cord dorsal horn. They are either released separately or co-released from presynaptic terminals of inhibitory neurons. Upon the binding to GABA_A _receptors and glycine receptors at postsynaptic membrane, they elicit inhibitory postsynaptic currents (IPSCs). IPSCs provide phasic inhibition in neuronal network and are important for information processing. In addition to its action at synaptic sites, recent studies in several brain regions of matured animals have indicated that low concentrations of ambient GABA can activate high affinity GABA_A _receptors that are expressed at extrasynaptic sites to elicit a sustained inhibitory current [1-5]. A term 'tonic inhibitory currents' has been used to describe this sustained inhibitory current [6]. Functionally, tonic GABAergic inhibition has been shown to control neuronal excitability in the brain [7-9]. Tonic inhibitory currents have been identified in rat cerebellar granule cells [1], granule cells of the dentate gyrus [2], thalamocortical relay neurons of the ventral basal complex [3], layer V pyramidal neurons in the somatosensory cortex [4], inhibitory interneurons in the CA1 region of the hippocampus [5]. However, not all CNS neurons that were examined displayed tonic inhibitory currents under normal conditions. For example tonic inhibitory currents were normally not observed in hippocampal pyramidal cells in brain slices from adult animals [[5,10], but see [11]].

The lamina II of the spinal dorsal horn (substantia gelatinosa) plays an important role in processing nociceptive input from fine myelinated Aδ – and unmyelinated C-primary afferents from the periphery [12]. In this area, inhibitory neurons produce feedback and feed-forward inhibition to control nociceptive input, and a reduction of inhibitory activity in lamina II can result in central sensitization [13,14]. Previous studies on inhibitory controls in the spinal cord dorsal horn have been mainly focused on inhibitory postsynaptic currents, i.e. phasic inhibition. Little is known about whether tonic inhibitory currents are present in this region and if so, whether tonic inhibitory currents are mediated by GABA receptors and/or glycine receptors.

## Materials and methods

The methods for preparing thick adult mouse spinal cord slices, as well as blind whole-cell patch-clamp recording techniques, have been described previously [15]. In brief, transverse spinal cord slices (500–600 μm in thickness) were prepared from L5 spinal cords of adult mice (Harlan, IN, USA) aged between 6 and 9 weeks. In each experiment, a spinal cord slice was transferred to a recording chamber (volume of 0.5 ml). The slice was supported at the bottom by a nylon mesh in the recording chamber. The slice was superfused with Krebs solution at flow rate of 10 ml/min. The Krebs solution contained (in mM): NaCl 117, KCl 3.6, CaCl_2 _2.5, MgCl_2_, 1.2, NaH_2_PO_4 _1.2, NaHCO_3 _25, and glucose 11. The solution was equilibrated with 95% O_2 _and 5% CO_2_, maintained at room temperature (22°C), and the pH of the Krebs solution was 7.35. Under a dissecting microscope with 40× magnification, lamina regions were identified based on morphological features. The lamina II was clearly discernible as a relatively translucent band across the superficial dorsal horn. Under visual guidance, the patch electrode was inserted vertically into the lamina II.

Whole-cell patch-clamp recordings were made from lamina II neurons with electrodes (5~10 MΩ) filled with an internal solution containing (in mM): K-gluconate 120, KCl 20, MgCl_2 _2, Na_2_ATP 2, NaGTP 0.5, HEPES 20, EGTA 0.5, and pH 7.2 adjusted with NaOH. Signals were amplified and filtered at 2 kHz and sampled at 5 kHz (Axopatch 200B). Spontaneous inhibitory postsynaptic currents (sIPSCs) were recorded with cells being held at 0 mV. Each recording was performed on a cell in a fresh slice without prior application of any agonist or antagonist. Isolation of GABAergic sIPACs was accomplished by including 2 μM strychnine in the bath solution. Tonic inhibitory currents were revealed following the application of 20 μM bicuculline for a period of 3 min. All compounds tested were applied through the bath solution at a flow rate of 10 ml/min. Analysis of sIPSCs, including threshold setting and peak identification criteria, were performed according to a method previously described [15]. Decay time constant (τ) of sISPCs was analyzed using Clampfit 9 (Axon Instruments, Inc., Sunnyvale, CA, USA). sIPSC frequency, amplitude, and average charge transfer (Q_sIPSCs_, integrated area under sIPSCs) were analyzed using Mini Analysis Program (Synaptosft, Inc., Decatur, GA, USA). Charge transfer (Q_PC_) associated with sIPSCs in a given time (t) was calculated using the equation Q_PC _= f × Q_sIPSCs _× t, where f is the frequencies (Hz) of sIPSCs, Q_sIPSCs _is the average charge transfer per sIPSC during a 60-s baseline recording, and t is the duration (60 s), respectively. The charge transfer associated with tonic currents was calculated according to the equation: Q_TC _= I_TC _× t, where Q_TC _is the charge transfer produced by tonic currents, I_TC _is the current amplitude at steady-state, and t is time (60 s). Unless otherwise indicated, data represent Mean ± SEM, Student's *t*-tests were used for statistical comparison, and significance was considered at the *P *< 0.05 level.

## Results

**Figure 1:**
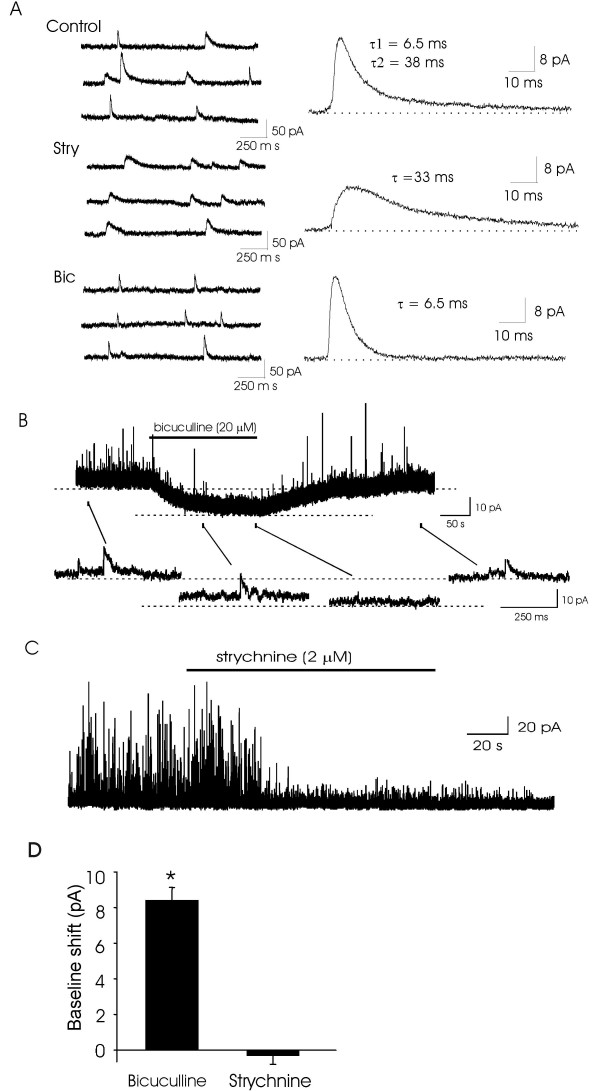
**Revealing tonic inhibitory currents in lamina II neurons of adult mice**. A). Traces on the left side show sIPSCs recorded in normal bath solution (control), following the applications of either 2 μM strychnine (Stry) or 20 μM bicuculline (Bic). Traces on the right side are the average of 100 sIPSCs. τ is time constant for averaged trace. B). Sample trace (top panel) shows the shift of baseline holding current following the application of 20 μM bicuculline. The lower traces show at an expanded scale the baselines before and during bicuculline application as well after wash off bicuculline. C). Sample trace shows that application of strychnine (2 μM) inhibited some sISPCs but did not affect baseline holding current. D). Bar graph shows pooled results of baseline shift following the application of either 20 μM bicuculline (n = 30) or 2 μM strychnine (n = 6). Bicuculline or strychnine was applied for 3 min in each experiment.

Under our voltage-clamp condition with cells being held at 0 mV, spontaneous inhibitory postsynaptic currents (sIPSCs) recorded from lamina II neurons were outward currents, and spontaneous excitatory postsynaptic currents (sEPSCs) were not detectable because the holding potential of 0 mV is at the reversal potential for sEPSCs. Phasic inhibitory activities (or sIPSCs) showed three types, rapid, slow, and mixed types, based on the kinetics of their decay phases. When these different types of sIPSCs are integrated together, it yields an sIPSC that best fits into a two-exponential equation (Figure 1A). Following the application of 2 μM strychnine to block glycine receptors, rapid type of sIPSCs disappeared but slow sIPSCs remained. When bath solution contained 20 μM bicuculline but not strychnine, only rapid type of sIPSCs could be observed (Figure 1A). All sIPSCs could be completely blocked in the presence of both bicuculline (2 μM) and strychnine (20 μM, not shown).

Application of 20 μM bicuculline not only inhibited slow types of sIPSCs, but also caused a shift of baseline holding current (Figure 1B). The shift of baseline hold currents represents the presence of tonic inhibitory currents mediated by GABA_A _receptors [6]. The amplitudes of the tonic inhibitory currents revealed by bicuculline were 8.4 ± 0.7 pA (n = 30, Figure 1D). After washing off bicuculline, hold currents returned to the baseline levels.

Glycinergic inhibitory postsynaptic currents contributed to phasic inhibitory activity in lamina II region, and application of glycine receptor antagonist strychnine (2 μM) blocked glycinergic sIPSCs. However, there was no significant change of baseline holding currents following the application 2 μM strychnine (Figure 1C). The baseline holding currents were -0.3 ± 0.5 pA (n = 6) in lamina II, not significantly different from the baseline noise level (Figure 1D). These results suggested that glycine receptors did not significantly account for the tonic inhibitory currents in dorsal horn lamina II neurons of adult mice.

**Figure 2:**
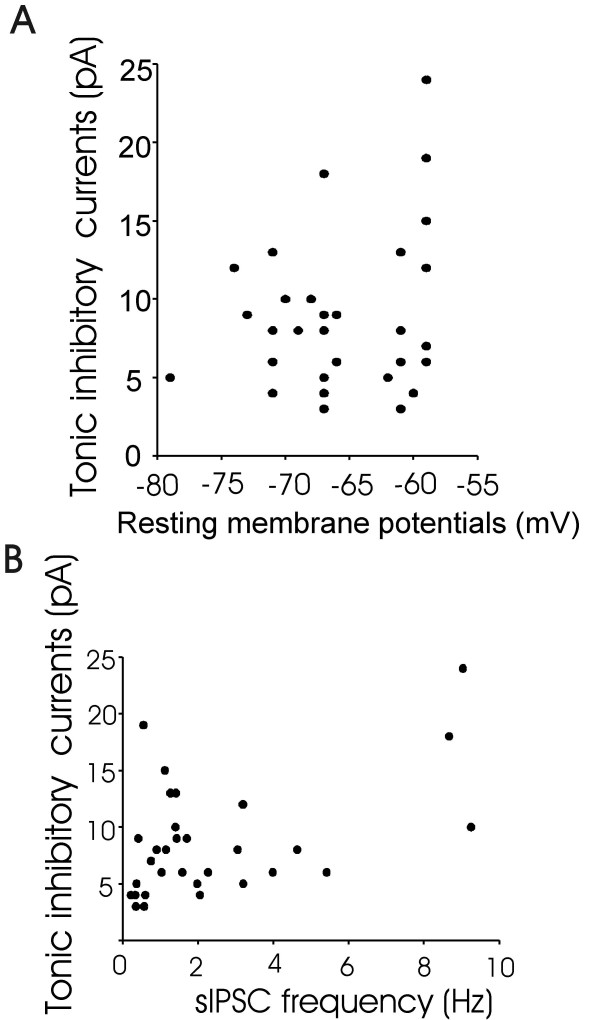
**Lack of correlation between tonic inhibitory currents and resting membrane potentials as well as between tonic inhibitory currents and total sIPSC activity**. A). Graph shows a plot of tonic inhibitory currents against membrane potentials for each recorded neuron (n = 30). B). Graph shows a plot of tonic inhibitory current against sIPSC frequency (n = 30). Tonic inhibitory currents were revealed by the applications of 20 μM bicuculline. Both resting membrane potentials and sIPSCs were measured before the application of bicuculline.

Amplitude of tonic inhibitory currents showed large variations among different recordings. One possible cause of this variation might be due to the differences of resting membrane potentials of these neurons. Therefore, we measured resting membrane potentials of each recorded neurons and plotted amplitudes of tonic inhibitory currents against resting membrane potentials of each recording. No relationship was found between tonic inhibitory current amplitudes and resting membrane potentials (r = 0.27, n = 30, Figure 2A). We determined whether amplitude of tonic inhibitory currents were in proportional to inhibitory synaptic inputs by plotting tonic inhibitory current amplitude against sIPSC frequency. We also did not find a strong association between the amplitudes of tonic inhibitory currents and the frequency of total sIPSCs (r = 0.5, n = 30, Figure 2B).

**Figure 3:**
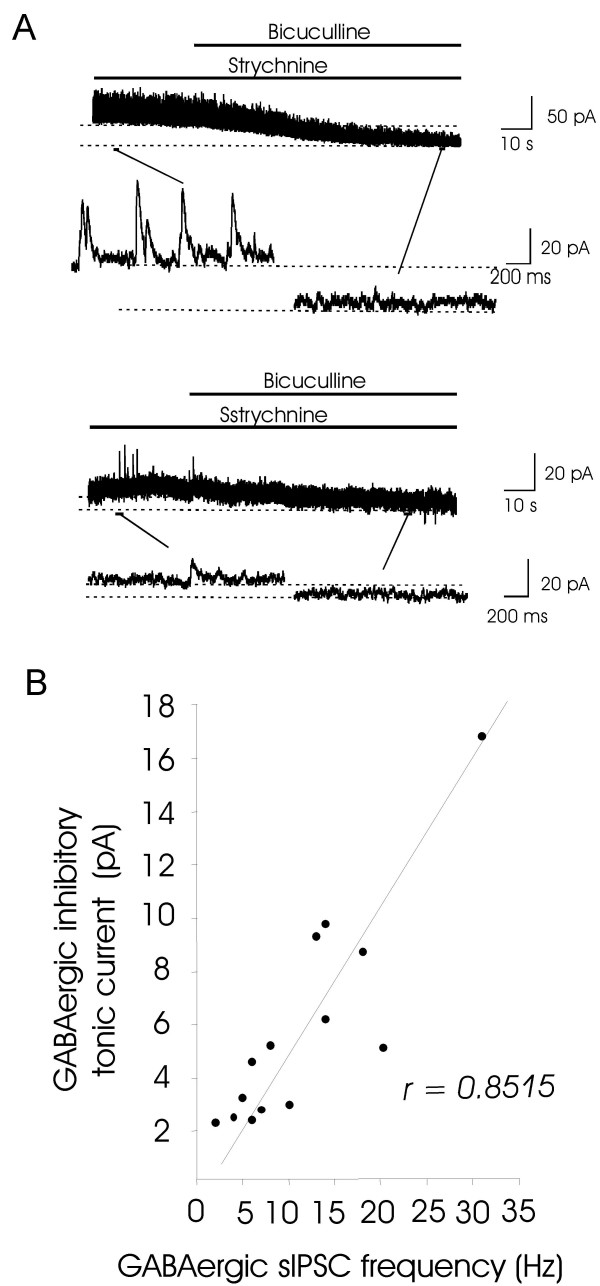
**Correlation between tonic inhibitory currents and GABAergic inhibitory synaptic activity**. A). Sample trace shows bicuculline-induced baseline shift in a lamina II neuron with high GABAergic inhibitory synaptic activity. B). Sample trace shows bicuculline-induced baseline shift in another lamina II neuron with low GABAergic inhibitory synaptic activity. C). Plot of GABAergic tonic inhibitory currents against frequency of GABAergic sIPSCs (n = 14). Linear regress coefficient (r) = 0.8515. All experiments were performed in the presence of 2 μM strychnine.

Total sIPSCs in the spinal cord dorsal horn included both glycinergic and GABAergic inhibitory synaptic activity. We isolated GABA_A _receptor-mediated sIPSCs from total sIPSCs by including 2 μM strychnine in bath solution. Under this condition, glycine receptor-medicated sIPSCs were completely abolished. We then applied 20 μM bicuculline to reveal tonic currents, and we found that neurons receiving higher frequency of GABAergic synaptic inputs usually had larger amplitudes of tonic inhibitory currents (Figure 3A), and neurons receiving lower frequency of GABAergic synaptic inputs usually had smaller amplitudes of tonic inhibitory currents (Figure 3B). There was a good linear relationship between the amplitude of GABAergic tonic inhibitory currents and the frequency of GABAergic IPSCs (r = 0.8515, n = 14, Figure 3C).

**Figure 4:**
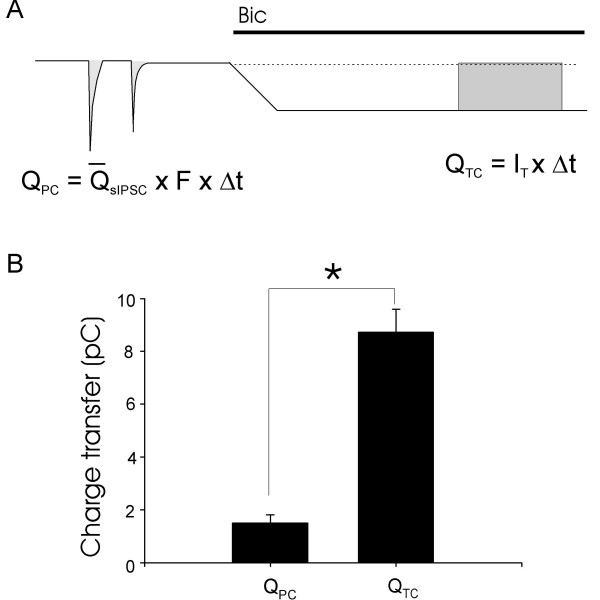
**Charge transfers mediated by phasic inhibitory currents and by tonic inhibitory currents**. A). Diagram illustrates the measurement of phasic inhibitory current charge transfer (Q_PC_) and tonic inhibitory current charge transfer (Q_TC_). B). Bar Graph shows the comparison between Q_PC _and Q_TC _(n = 32). Tonic currents were revealed by the application of 20 μM bucuculline. Strychnine was not included in bath solution.

Charge transfer through GABA_A _receptors is a measure of inhibition for both phasic and tonic inhibitory currents. We determined charge transfer carried by phasic inhibitory currents and by tonic inhibitory currents in a period of 60 sec recording (Figure 4A). Charge transfer was 1.5 ± 0.31 pC (n = 44) for the total phasic currents mediated by both GABAergic and glycinergic inhibitory postsynaptic currents (Figure 4B). On the other hand, charge transfer was 8.7 ± 0.89 pC (n = 44) for tonic inhibitory currents mediated by GABA_A _receptors, and was about 6 times of the total charge transfer mediated by both GABAergic and glycinergic inhibitory postsynaptic currents (Figure 4B).

## Discussion

Using spinal cord slice preparations from adult mice, the present study shows that tonic inhibitory currents are present in lamina II of the dorsal horn and was solely mediated by GABA_A _receptors, that the extent of tonic inhibition is proportional to GABAergic inhibitory synaptic activity, and that tonic currents transfer charges substantially higher than phasic currents. The results provide new information about inhibitory activities in a nociceptive processing region.

We have observed a large variation of the size of tonic inhibitory currents. It has been suggested that ambient GABA concentrations around the extrasynaptic domains of neurons is a factor that determines the size of tonic inhibitory currents. The concentrations of ambient GABA vary from tens of nanomolar to a few micromolar based on in vivo microdialysis studies in the brain [16-19]. One possible cause of the variation may be regional and temporal differences in GABAergic neuron activity. We have shown that GABAergic sIPSC frequencies recorded in lamina II neurons have a big variation, and GABAeric sIPSC frequency and tonic current size are correlated. It is very likely that neurons with higher GABAergic sIPSC frequency have higher concentrations of ambient GABA around them due to more frequent spillover of GABA from inhibitory synapses. Therefore, these neurons have larger-sized tonic inhibitory currents mediated by extrasynpatic GABA_A _receptors. This is consistent with previous studies in cerebellar granule cells, which shows that ambient GABA concentration is maintained by action potential-dependent vesicular release and is responsible for tonic GABA_A _receptor activation [1,7,20-23].

Although phasic inhibition is mediated by both GABA_A _and glycine receptors in spinal cord lamina II of adult mice, we did not observe any contribution of glycine receptors to tonic inhibitory currents. The lack of glycine receptor-mediated tonic inhibitory currents could be mainly due to the lack of high affinity glycine receptor isoforms. Of different glycine receptor isoforms (α1β, α2β, α3β) identified in the CNS, their EC50 values for glycine were normally above 50 μM [24]. In contrast, extrasynaptic GABA receptors that contribute to tonic currents were found to be high affinity isoforms (e.g. α_6_β_x_δ, α_4_β_x_δ and α_5_β_x_γ_2_) with EC50 in nanomolar range [6].

The large charge transfer carried by GABAergic tonic inhibitory currents shown in this study indicates a persistent increase in the input conductance of lamina II dorsal horn neurons. The increase of input conductance in neurons can decrease the size and duration of the excitatory postsynaptic potentials (EPSPs), and make neurons less likely to generate action potentials. Indeed, tonic currents have been shown to be a critical determinant that controls neuron excitability in cerebellar granule cells [7-9]. In spinal cord lamina II, decreases of neuron excitability by GABAergic tonic inhibitory current may be an important mechanism to control nociceptive inputs and to prevent central hyper-sensitization.

## Competing interests

The author(s) declare that they have no competing interests.
